# Sequence motif finder using memetic algorithm

**DOI:** 10.1186/s12859-017-2005-1

**Published:** 2018-01-03

**Authors:** Jader M. Caldonazzo Garbelini, André Y. Kashiwabara, Danilo S. Sanches

**Affiliations:** Department of Computer Science, Bioinformatics Graduate Program, Federal University of Technology - Paraná, Cornélio Procópio, PR Brazil

**Keywords:** Motif, Evolutionary algorithms, Memetic algorithms, Heuristics, Transcription factor binding sites

## Abstract

**Background:**

*De novo* prediction of Transcription Factor Binding Sites (TFBS) using computational methods is a difficult task and it is an important problem in Bioinformatics. The correct recognition of TFBS plays an important role in understanding the mechanisms of gene regulation and helps to develop new drugs.

**Results:**

We here present Memetic Framework for Motif Discovery (MFMD), an algorithm that uses semi-greedy constructive heuristics as a local optimizer. In addition, we used a hybridization of the classic genetic algorithm as a global optimizer to refine the solutions initially found. MFMD can find and classify overrepresented patterns in DNA sequences and predict their respective initial positions. MFMD performance was assessed using ChIP-seq data retrieved from the JASPAR site, promoter sequences extracted from the ABS site, and artificially generated synthetic data. The MFMD was evaluated and compared with well-known approaches in the literature, called MEME and Gibbs Motif Sampler, achieving a higher f-score in the most datasets used in this work.

**Conclusions:**

We have developed an approach for detecting motifs in biopolymers sequences. MFMD is a freely available software that can be promising as an alternative to the development of new tools for *de novo* motif discovery. Its open-source software can be downloaded at https://github.com/jadermcg/mfmd.

**Electronic supplementary material:**

The online version of this article (doi:10.1186/s12859-017-2005-1) contains supplementary material, which is available to authorized users.

## Background

Sequence motifs are small sequences capable of acting as binding sites for a particular transcription factor [1]. In many situations, the localization of the motifs should be learned without prior knowledge. For that reason, this problem is called *de novo* motif discovery [2].

Transcription factors are specific proteins that bind to distinct sites on the genome. This binding is an essential process in gene regulation which may lead to changes in transcriptional activity for a particular gene target [3]. These sites are short (< 30 bps) and have a typical nucleotide sequence, although there may normally be variations due to mutations that occurred because of the selective pressure that the genome has undergone over time [4].

According to [5], several approaches have been proposed to solve efficiently this problem. Also, we have highlighted in this work the probabilistic and exact approaches [6].

Probabilistic methods try to maximize the relative entropy or Kullback-Leibler divergence [7], obtained from the construction of a Position Specific Score Matrix (PSSM). There are several algorithms within this set of which include: MEME [8], CONSENSUS [9] and Gibbs Motif Sampler [10]. These algorithms usually have a quick run time. However, they may be “stuck” in *local optima*.

Exact approaches usually use the consensus sequence for motif representation, employing some mathematical optimization as the search model. In general, these algorithms have a high convergence time, in particular for long motif length [11]. In contrast, they may escape from local optima due to the exact nature of his search. Examples include SPELLER [12] and WEEDER [13].

In this paper, we introduce MFMD a memetic algorithm [14] whose goal is to solve *De novo* motif discovery problem. MFMD uses a modified version of the Greedy Randomized Adaptive Research Procedure (GRASP) [15] to build an initial population of solutions. In addition, we have included the Variable Neighborhood Search (VNS) algorithm [16], that is a greedy local search method that explores the solution space through systematic exchanges of increasingly distant neighborhood structures. Also, the VNS step is important for recombination and mutation sub-stages to fine-tune individuals previously constructed by GRASP.

### Previous work

We have developed in previous work two approaches called Discovery Motifs by Evolutionary Computation (DMEC) [17] and Discovery Motifs by Memetic Algorithms (DMMA) [18]. In the DMEC, we evolved a population of PSSM matrices using a canonical evolutionary algorithm and a greedy mutation operator. Good results were obtained in several synthetic datasets and some real ones, such as the cyclic-AMP dataset (CRP). DMMA is an evolution of DMEC where we have some heuristics along with traditional evolutionary algorithm. Furthermore, the DMMA algorithm obtained a substantial gain compared to DMEC. MFMD extends the idea of DMMA and DMEC including a new mechanism of search that control the exploration vs exploitation in the search space.

For most of these approaches, the emphasis is on the application of canonical evolutionary algorithms to solve biosequence problems. Our motivation is slightly different in that we intend to use the flexibility of evolutionary algorithms in addition to the efficiency that some heuristics have. Thus, it was possible to develop strategies that are more applicable to the resolution of discovery motif problems in real situations.

### Problem definition

Although there are several formulations of this problem, we will begin with the canonical and more general definition of motif discovery in the following manner.

Let *S*={*s*_1_,*s*_2_,⋯,*s*_*n*_} be the set of sequences over *Σ*={*A,C,G,T*} and let *w* be the motif length. In this paper, we assume that the length of all sequences is equal to *L* and 0<*w*≪*L*.

The problem consists in finding the most promising pattern of subsequences *X*^∗^={*x*_1_,*x*_2_,…,*x*_*n*_} of size *w* and their respective initial positions in each sequence in *S*. The choice of a particular pattern is based on the definition of one or more score functions that measure the similarity or difference between the motifs pattern and their respective occurrences. Li et al. (1999) proved that the canonical definition of motif problem is NP-Hard even with the most simplified assumptions [19].

There are several methods for measuring the quality of the motifs. The objective functions should be able to reflect the efficiency of a modeling accurately. An inadequate evaluation function will not be able to provide a good solution even whether a strong optimization algorithm is used. We have used in this work the Information Content Score [20] and the Complexity Score [21] as objective functions.

Information Content (IC) can be interpreted as an energy estimate that a set of motifs exerts on its respective binding site as opposed to the rest of the organism’s genome [1]. In other words, the IC measures the statistical difference between a motif from a specific probabilistic model or a motif from a background probabilistic model (usually inferred from the genomic sequences of a given organism). The background statistical model is typically constructed under a homogeneous Markov chain of order zero or higher. Complexity score was defined by Gary B. Fogel and Weekes [21] and penalizes sequences with low complexity, i.e., whose entropy value is very low. In general, this may disrupt the search and should be considered a noise [22].

## Implementation

**Fig. 1:**
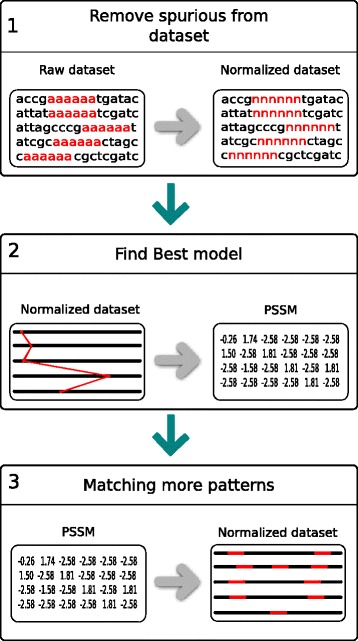
MFMD pipeline. (1) In Preprocessing step MFMD uses DUST to remove sub-sequences with low complexity entropy. If DUST can not be run, MFMD uses an objective function defined in [21] to mitigate this problem. (2) In Pattern Discovery step, MFMD attempts to find the best PSSM matrix using GRASP and VNS heuristics. (3) In Pattern Matching step, MFMD uses the PSSM matrix found in the previous step to predict the initial positions of the motifs in the dataset

MFMD was developed using Java programming language release 8u111 (64-bit) and Ubuntu Linux operating system. The algorithm evolves a population of PSSM matrix and finds solutions that maximize the Information Content Score and Complexity Score using a bi-objective Weighted Sum Model. The algorithm receives as input a typical DNA dataset of co-regulated genes and returns the initial positions of the found motifs. MFMD was divided into three steps: Pre-Processing, Pattern Discovery and Pattern Matching. Figure 1 illustrates the simplified MFMD pipeline.

### Preprocessing

This step aims to find and remove subsegment entries that can direct the search to invalid locations. According to D’haeseleer [6] these subsequences, called spurious [23], can contribute negatively to the performance of the search algorithms. To mitigate this problem, before the algorithm starts the pattern discovery phase, we execute DUST [24], to meet the above requirements. DUST is a tool created by R. L. Tatusov and D. J. Lipman, whose objective is to remove sub-sequences with low complexity from the dataset.

### Pattern discovery

This step consists of optimizing and discovering the best PSSM matrix from an input dataset. Moreover, we have sub-divided the cycle into Initial Population Construction, Fitness Calculation, Recombination, Mutation and Selection steps.

#### Initial population

This step is the most important action of the algorithm in which each solution is represented by a tree-like data structure. In this structure, the nodes represent the initial positions, where the root node represents the initial position of the first dataset sequence. In this way, the algorithm creates a tree solution for each valid position in the dataset sequence. For example, whether the dataset has 100 valid positions, then the algorithm will generate 100 trees, each with its starting position.

The total number of valid positions can be obtained by Equation *v*=*L*−*w*+1 where *v* is the total number of valid positions, *L* is the size of each sequence and *w* is the size of a particular motif. In MFMD, solutions are built gradually with the aid of a GRASP-based heuristic. In general, this paradigm shift led the initial solutions to the most promising locations in the search space.

The modifications consist in the use of a variable *q* that modifies the algorithm behavior and determines whether it will make a greedy or a random choice. The multi-start function has also been disabled because in this approach GRASP is only used as a startup tool. Then, at each iteration, a number *n*∈ [0,1] is uniformly drawn, and the behavior of the algorithm follows Eq. 1:

1 $$ choice=\left\{ \begin{array}{ll} greedy, & n \leq q.\\ random, & \text{otherwise}. \end{array} \right.  $$

If the choice is greedy, the algorithm tests whether there are still other positions having a score equal to the best score found so far, i.e., whether there is a tie between the scores from the valid positions list. If so, all tied positions are added to the tree. If the choice is not greedy, the solutions are ranked in a Restricted Candidate List (RCL). Then a solution of the list is uniformly chosen and included in the tree.

The *RCL* size and the *q* parameter can be quite different, but in our experiments we have found the best values empirically, hence, we have used the following values *RCL*=5 e *q*=0.9. Finally, the algorithm is done when all initial positions are included in the tree, i.e, when the height of the tree is equal to the number of sequences minus one.

The algorithm complexity grows according to the size of the dataset. For example, whether a dataset has *N* sequences of length *L*=30 and motifs with length *w*=5 there will *L*+*w*−1=26 valid positions in that dataset. Thus, the algorithm will make 26^2^ comparisons between the first and second sequences, plus 26^2^ comparisons between the second and third sequences, and so on.

Therefore, the final complexity of the algorithm is *O*((*L*−*w*+1)^2^×*N*−1) which can be summarized in *O*(*N*×*L*^2^). In the worst case the algorithm can achieve the complexity *O*(*L*^*N*^) whether all valid positions of all dataset sequences should tie in terms of score value. However, this is extremely unlikely and in practice, we have only a few draws occurring at each iteration with complexity *O*(*N*×*L*^2^) prevails.

The objective of this approach is to establish a compromise between the need to maintain the practical computational algorithm and the desire to obtain the mathematically optimal alignment.

#### Fitness calculation

Fitness is calculated by converting the initial positions of each individual into a structure called Multiple Local Sequence Alignment (MLSA). From the MLSA it is possible to calculate the Position Specific Score Matrix (PSSM). The PSSM is a zero-order non-homogeneous Markov chain [7] commonly used to represent probabilistic models of motifs whose statistical independence between the different “columns” of an MLSA is assumed. That means, from a statistical point of view, the nucleic bases that form the regulatory elements do not correlate. In practice, according to Benos et al., this independence is a good approximation [25].

For a motif of size *w*, a PSSM takes the form of a matrix 4×*w*. More details can be reviewed at [26]. The fitness of each individual was calculated using the bi-objective weighted sum model, whose functions were: Information Content (Eq. 2) and Complexity Score (Eq. 3).

2 $$ \text{IC} = \sum\limits_{i=1}^{\varSigma} \sum\limits_{j=1}^{w} \ \Theta_{(i,j)} \log_{2} \left[\frac{\Theta_{(i,j)}}{\Theta_{(0,i)}}\right]  $$

Where *w* is the motif size, Σ is the number of letters from the alphabet (*Σ*=4 for nucleotides), *Θ*_(*i,j*)_ is the matrix of the relative frequencies and *Θ*_(0,*i*)_ is the vector of background probabilities. The IC measures the statistical difference between a motif from a specific probabilistic model or a motif from a probabilistic background model [1]. The specific probabilistic model is constructed using a non-homogeneous Markov chain of order 0 or higher. In particular, we use the PSSM model that has zero order. The background statistical model is typically constructed under a homogeneous Markov chain of order zero or higher.

3 $$ \text{CS} = \sum\limits_{j=1}^{\Sigma} \sum\limits_{j=1}^{w} \log_{N}\left[\frac{w!}{\varPi n_{i}!}\right]  $$

Where Σ is the number of letters from the alphabet. (*Σ*=4 for nucleotides), *N*=4 for nucleotides, *w* is the motif size and *n*_*i*_ is the total number of nucleotides *i*∈*A,C,G,T*. The Complexity Score was defined by Gary B. Fogel and Weekes [21] and penalizes low complexity sequences, i.e., sequences whose entropy value is very low. In general, this may disrupt the search and should be considered a noise [22]. For example, the motif “aaaaaa” (*n*_*a*_=6,*n*_*c*_=0,*n*_*g*_=0,*n*_*t*_=0) will have minimal complexity since it will obtain a maximum value in π*n*_*i*_. On the other hand, the motif “atacgt” (*n*_*a*_=2,*n*_*c*_=1,*n*_*g*_=1,*n*_*t*_=2) will obtain a value of complexity greater than the previous one, since the value of the function π*n*_*i*_ will be smaller. In this example, $CS(aaaaaa) = \frac {6!}{6 \times 6 \times 6 \times 6 \times 6 \times 6} = \frac {720}{46656} = 0.0154$ and $CS(atacgt) = \frac {6!}{2 \times 2 \times 2 \times 1 \times 1 \times 2} = \frac {720}{16} = 45$.

The total fitness of each individual is defined by the Eq. 4:

4 $$ Fi = v IC + (1 - v) CS  $$

In Eq. 4, *v*∈ [0,1] are arbitrary weight chosen in a random way. The parameter *v* changes the importance level of each objective function. In our experiments *v*=0.8 is the value that produced the best results. This equation establishes a relation between the objective functions and the parameter *v*. In particular, it becomes important when is not possible to remove spurious a priori.

Since DUST runs in the preprocessing step, the MFMD can be reduced to a mono-objective algorithm running only the Information Content Score. This brings faster execution and does not compromise the accuracy of the approach. For palindromic sequences, the reverse motif complement must also be taken into account. Whether the motif is a palindrome, this predilection may lead the algorithm to more accurate results. It is important to note that when inserting the reverse complement in the score calculation, the PSSM matrix becomes a symmetric matrix.

#### Recombination, mutation and selection

The recombination operator is applied in some individuals from the initial population *P*. The individuals are selected in a random way. Also, the recombination occurs between pairs called individuals parents generating child individuals that are stored in an new population called intermediate population *Q*. At each recombination, the algorithm calculates the scores of parents *p*_1_ and *p*_2_, selects the best and puts it in *p*^∗^. After the children *c*_1_ and *c*_2_ are generated, The score of these are also calculated and compared with *p*^∗^. If *F*(*c*_1_)<*p*^∗^ then the mutation occurs through the local search in *c*_1_ using the VNS heuristic. The same situation holds true for the child *c*_2_. The mutation is performed through the following rule (Eq. 5):

5 $$ child=\left\{ \begin{array}{ll} VNS(child), & F(child) < p^{*}.\\ child, & \text{otherwise}. \end{array} \right.  $$

After mutation operator, populations *P* and *Q* are joined generating the *R* population (*R*=*P*∪*Q*). Then the *R* population is sorted in descending order and the first |*P*| solutions from *R* are put back to the population *P*.

### Pattern matching

This step consists in the application of statistical techniques for the motifs recognition that were not found along the Pattern Recognition stage. In many cases, the promoter regions have more than one binding site. Therefore, it is expected that search algorithms will be able to find as many motifs as possible from a particular co-regulated gene.

The MFMD assume the distribution of the final scores is a Gaussian distribution [27] of mean *μ* and standard deviation *σX*(∼*N*(*μ*,*σ*^2^)). The parameters of the statistical model were estimated using the PSSM matrix found in the previous step. Thus, the scores are normalized and transformed into z-scores using Eq. 6:

6 $$ z = \frac{x - \mu}{\sigma}  $$

Where *x* is the raw score, *μ* is the mean and *σ* is the estimated standard deviation.

Then the *p*-value is calculated using the cumulative distribution function defined by Eq. 7:

7 $$ F_{X}(x) = \int_{-\infty }^{x}f_{X}(t)~ dt  $$

Where *F*_*X*_(*x*)=*P*(*X*≤*x*) or *P*(*a*<*X*<*b*)=*F*_*X*_(*b*)−*F*_*X*_(*a*), where *b*=1 e *a*=*x*.

**Fig. 2:**
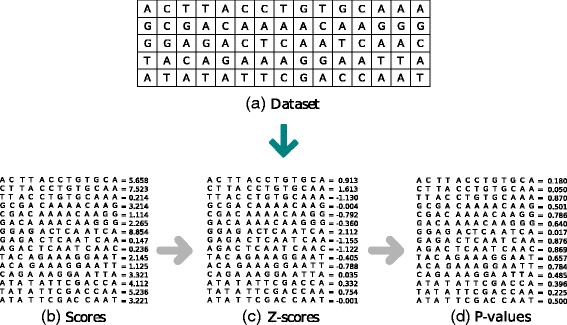
**a** Sequence dataset. **b** Splitting the dataset into *w*−*mers* (*w*=13). For each window, the score is calculated using the PSSM matrix found in Pattern Discovery step. **c** Transformation of the scores in z-scores. **d** The *p*-values are calculated from the z-scores. A cut-off point can be used to sort new motifs

The objective is to calculate the area under the curve and find which positions have the highest statistical significance. In short, the following actions are performed in this step (Fig. 2): (1) Split the entire dataset into fragments of size *w*; (2) Calculate the probability of each fragment using the probabilistic model found in the Pattern Discovery step, i.e., calculate the *Pr*(*seq*|*Model*); (3) Normalize the scores and turn them into z-scores; (4) Calculate the cumulative distribution function (FDA) for each z-score; (5) Choose only the values that satisfy significance level (ex. 0.0001) previously set by the user.

### Illustrative examples

Let us consider the dataset *S*={*seq*_1_,*seq*_2_} of length *L*=180 from the alphabet *Σ*=*A,C,G,T*. In addition, we have a motif size *w*=11. There are *L*−*w*+1=170 valid positions and, for each of them, MFMD constructs a different solution tree. Without loss of generalization and for simplification purposes we will consider that the dataset is normalized and therefore we will use Eq. 2 to calculate the scores.

**Fig. 3:**
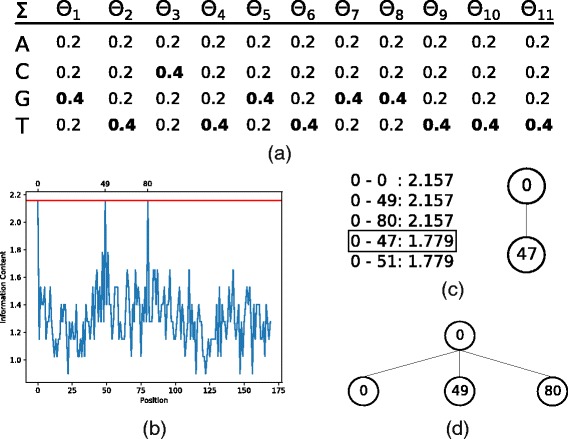
**a** Motif parameters were calculated using uniform background probability *Pr*(*a*)=*Pr*(*c*)=*Pr*(*g*)=*Pr*(*t*)=0.25 and *pseudocounters*=1**b** Information Content calculated using motif parameters. **c** Semi-greedy insertion. **d** Greedy insertion

Here, we introduce how the tree is generated from the first valid position (*GTCTGTGGTTT*) whose parameters are represented by *Θ* and can be viewed in the Fig. 3a.

>seq1

GTCTGTGGTTTtttccgtaaacccaacacaaacaaaccctccgcc

gtgaaacggtggcccccgatcaagtggggtctatgaagttatgtg

agcggagcgtaatatagcgtatacaactagatcaccttgtgcagt

gtgattccgccctctcctggctctctctcgtcgtgggcatatgtt

>seq2

gtctgtggtgtacttgcataaccggatcttcaaccatctcgagga

cggtgtgtgtggtttttccgattagagggttaggtgtcagtggtt

tgctttctaattgatttacgatatatggatcctggacacacacac

tgtaatacttggtggatgccccggatgttaaggatggcgcacatt

The building of the solutions tree depends on a random number *n*∈ [0,1] and a constant *q*=0.9. Whether *n*≤ *q* the algorithm constructs the tree greedily, otherwise it uses a Restricted Candidates List *RCL*=5 to choose the next node.

Whether the choice is greedy, only the node with the best score is added to the tree. If there is a tie between the best node and the others, all tied nodes are added to the tree. Figure 3b illustrates a model where three nodes (0,49 and 80) have the same score (2≤*s*≤2.2). In this instance all three nodes would be added to the tree, as shown in Fig. 3c.

If the choice is semi-greedy, the nodes are sorted in descending order and the top five are added in the RCL. Then, a node is uniformly chosen to compose the tree as shown in Fig. 3d. It is interesting to note that if the choice is greedy, more than one node can be added to the tree whereas in semi-greedy choice only one node is added.

### Datasets description

The following datasets were used in this paper: (1) Simulated data: datasets and motifs algorithmically generated; (2) JASPAR: datasets and motifs extracted from the JASPAR site [28]; (3) ABS: datasets and motifs extracted from the ABS site [29]; (4) SCPD: datasets and motifs extracted from the SCPD site [30]; (5) CRP: dataset and motifs extracted from the publication of Stormo and Hartzell [20].

It is important to highlight that the ABS, SCPD, CRP and JASPAR datasets have real background data and motifs. For simplicity, at this point we will call the ABS, SCPD and CRP datasets of real datasets experiments. We have emphasized the discussion only in ABS, SCPD, CRP and JASPAR datasets since they are real, publicly available and they have been used extensively in other works.

In JASPAR were randomly selected the datasets based on their identification. Five datasets were chosen using data collected from ChIP–seq experiments. Table 1 shows a summary of these datasets. Finally, eleven datasets were used in real datasets experiments, seven extracted from the ABS site [29], three extracted from the SCPD site [30] and one extracted from the publication of Stormo and Hartzell [20]. Table 2 shows the information about these datasets.

**Table 1:** Summary of JASPAR datasets

ID	Name	Species	Number of sequences
MA0003.2	TFAP2A	H. sapiens	5098
MA0036.2	GATA2	H. sapiens	4380
MA0037.2	GATA3	H. sapiens	4628
MA0050.2	IRF1	H. sapiens	1362
MA0150.2	NFE2L2	M. musculus	726

**Table 2:** Summary of real datasets experiments

ID	Name	Site	Number of sequences	Number of motifs
CREB	cAMP Response Element	ABS	17	19
HNF-1	Hepatocyte Nuclear Factor-1	ABS	22	27
MEF2	Myocyte Enhancer Factor-2	ABS	17	17
MyoD	Myogenic Differentiation-1	ABS	17	21
NF-kB	NF Kappa-Light-Chain-Enhancer	ABS	6	8
SRF	Serum Response Factor	ABS	20	36
TBP	TATA-Binding Protein	ABS	95	95
PDR3	Pleiotropic Drug Response	SCPD	7	18
REB1	RNA polymerase I enhancer	SCPD	15	20
MCB	Mlu I cell cycle boxes	SCPD	6	12
CRP	cAMP Receptor Protein	Stormo and Hartzell	18	24

For details and results about simulated datasets, see Additional file 1.

### Evaluation methods

For each dataset, 30 tests were performed and the results obtained were compared to two other approaches: Gibbs Motif Sampler [31] and Meme (Multiple EM for Motif Elicitation) [8].

To measure the performance of each strategy, we adopted the initial position that each approach found. A position is considered correct if it equals the real or varies two units more or less. For example, if the annotated position of a given motif is 60, all of these values will be considered correct: 58, 59, 60, 61 e 62. For each experiment performed by MFMD, we calculated the mean and standard deviation of the performance measures. For the experiments performed by Meme and Gibbs Motif Sampler, values were used which showed better execution performance.

We evaluated the approaches according to the metrics of information retrieval precision, recall, and f-score [32]. These measures have a minimum value of zero and a maximum value of one, where zero represents no predicted position, and one characterizes a perfect prediction.

#### Rank analysis

The results were compared using the dominance method proposed by L. I. Kuncheva and J. J. Rodríguez [33]. In this system, each approach receives a score when compared to the other approaches. The dominance hierarchy is determined by the classification of methodologies according to a score calculated through the losses and victories that each approach has achieved in each f-score measure. This corresponds to the total number of times that, for example, the “A” approach was able to be better than the “B” approach minus the total number of times that the “B” approach was better than the “A” approach.

In addition, wins and losses were defined in terms of the f-score values that each strategy was able to achieve. Since the f-score represents the harmonic mean between precision and recall, the magnitude of its value is directly influenced by both measurements, i.e., a low precision value will imply a low f-score even if the recall is high. The inverse relationship is also true.

#### Statistical analysis

The objective of this analysis was to compare the results obtained by the MFMD with the results achieved by the other approaches using statistical methods of hypothesis testing. The purpose of this test is to indicate if there is a significant difference between them and to determine which approach presented the best performance. Statistical significance tests were performed between the differences of the f-scores by all approaches.

The hypotheses to be tested were:

8 $$ \left\{ \begin{array}{ll} H_{0}: & \text{Samples approaches are draw}\\ & \text{from distributions with the same mean value}.\\ H_{1}: & \text{Samples approaches are draw}\\ & \text{from different distributions}. \end{array} \right.  $$

The analyzes consisted of the following steps: (1) sample selection: some datasets were selected to compare the statistical test. There were 2 of each synthetic group, 5 ChIP-seq and 2 real datasets experiment, totaling 21 datasets; (2) statistical analysis: the analyzed parameter was the f-score calculated from the 30 executions performed in each dataset by each algorithm; (3) the Shapiro-Wilk test [34] was applied to each set of parameters. In the case of normality being verified, a paired Student’s T test [35] was applied. Otherwise, the non-parametric test used was the Wilcoxon [36] paired; (3) the significance level used was 0.05 or 95%.

## Results and discussion

Tables 3 and 4 illustrate the results obtained by the predictors in JASPAR and real datasets experiments, respectively. It is important to note in Table 4, that in some datasets MEME obtained zero in precision, recall and f-score measures. In particular, MEME reached this value in datasets CREB and NFKB. Also, it is evident that the deviation measured by the initial positions predicted by MEME was higher than two, leading to the true positive (TP) counts to zero. Consequently, this led to the values of precision, recall and f-score also at zero.

**Table 3:** Results achieved by predictors in JASPAR datasets

Dataset	Predictor	Precision	Recall	F-Score
GATA2	MFMD	0.968±0.011	0.972±0.021	0.970±0.057
	MEME	0.948	0.948	0.948
	GIBBS	0.826	0.188	0.307
GATA3	MFMD	0.971±0.015	0.965±0.011	0.968±0.019
	MEME	0.965	0.965	0.965
	GIBBS	0.440	0.094	0.156
IRF1	MFMD	0.829±0.018	0.835±0.023	0.832±0.022
	MEME	0.903	0.903	0.903
	GIBBS	0.695	0.510	0.588
NFE2L2	MFMD	0.879±0.011	0.881±0.031	0.880±0.041
	MEME	0.866	0.866	0.866
	GIBBS	0.754	0.754	0.754
TFAP2A	MFMD	0.951±0.013	0.949±0.070	0.950±0.010
	MEME	0.515	0.515	0.515
	GIBBS	0.950	0.186	0.311

**Table 4:** Results achieved by predictors in real datasets experiments

Dataset	Predictor	Precision	Recall	F-Score
CREB	MFMD	0.647±0.024	0.578±0.044	0.611±0.031
	MEME	**0**	**0**	**0**
	GIBBS	0.529	0.473	0.500
CRP	MFMD	0.909±0.039	0.833±0.033	0.869±0.027
	MEME	0.904	0.791	0.844
	GIBBS	0.941	0.666	0.780
HNF1	MFMD	0.772±0.013	0.629±0.032	0.693±0.019
	MEME	0.136	0.111	0.122
	GIBBS	0.500	0.222	0.307
MCB	MFMD	0.999±0.030	0.667±0.042	0.800±0.030
	MEME	0.692	0.750	0.719
	GIBBS	0.750	0.750	0.750
MEF2	MFMD	0.700±0.033	0.823±0.030	0.756±0.024
	MEME	0.705	0.705	0.705
	GIBBS	0.176	0.176	0.176
MYOD	MFMD	0.363±0.016	0.380±0.024	0.372±0.018
	MEME	0.235	0.190	0.210
	GIBBS	0.208	0.238	0.222
NFKB	MFMD	0.667±0.040	0.500±0.099	0.571±0.062
	MEME	**0**	**0**	**0**
	GIBBS	0.667	0.500	0.571
PDR3	MFMD	0.850±0.035	0.944±0.046	0.894±0.034
	MEME	0.653	0.944	0.772
	GIBBS	0.928	0.722	0.812
REB1	MFMD	0.800±0.027	0.600±0.025	0.685±0.021
	MEME	0.333	0.350	0.341
	GIBBS	0.266	0.200	0.228
SRF	MFMD	0.477±0.007	0.583±0.014	0.525±0.008
	MEME	0.440	0.611	0.511
	GIBBS	0.514	0.500	0.507
TBP	MFMD	0.657±0.004	0.768±0.008	0.708±0.006
	MEME	0.578	0.578	0.578
	GIBBS	0.308	0.347	0.326

Some predictors failed to score in these experiments because they found initial positions with a deviation greater than 2. These data are highlighted in bold

Table 5 shows the results obtained by the approaches in the ranking analysis. Moreover, it is possible to observe that MFMD presented a higher score (ranking) in relation to the other approaches compared for all datasets analyzed. The good relationship between precision and recall evidenced that the MFMD achieved a balance between the true positives and the predicted false positives.

**Table 5:** Wins and losses in JASPAR and real datasets experiments

Predictor	Dataset	Wins	Losses	Total
MFMD	JASPAR	9	1	8
	Real	21	0	21
MEME	JASPAR	6	4	2
	Real	5	17	–12
GIBBS	JASPAR	0	10	–10
	Real	6	15	–9

In Table 6 all approaches are ordered according to the performance obtained in Table 5. In this case, the leftmost algorithm indicates a better performance compared to the rightmost algorithm (ordering from best to worst). From the analysis of Table 6, we can verify that MEME performed well in the JASPAR datasets. This was even more evident in the data presented in Tables 3 and 7, where we highlight the good behavior of this algorithm in the GATA3 and IRF1 datasets. On the other hand, the Gibbs Motif Sampler has obtained good results in real datasets experiments. However, MFMD still figures first in both. This demonstrates the good capability of MFMD to handle datasets of varying sizes.

**Table 6:** Ranking of algorithms according to Table 5 (from best to worst)

JASPAR datasets:		
MFMD	MEME	GIBBS
Real datasets experiments:		
MFMD	GIBBS	MEME

**Table 7:** Statistical test between MFMD vs GIBBS and MFMD vs MEME approaches

Type	Group/Dataset	Approach	*P*-value	Result	Approach	*P*-value	Result
ChIP	GATA2	MFMD	2.2*e*−16	+	MFMD	1.327*e*−3	+
		GIBBS			MEME		
	GATA3	MFMD	2.2*e*−16	+	MFMD	0.1599	=
		GIBBS			MEME		
	IRF1	MFMD	2.2*e*−16	+	MFMD	2.200*e*−16	-
		GIBBS			MEME		
	NFE2L2	MFMD	2.2*e*−16	+	MFMD	0.0476	+
		GIBBS			MEME		
	TFAP2A	MFMD	2.2*e*−16	+	MFMD	2.200*e*−16	+
		GIBBS			MEME		
Real	SRF	MFMD	3.736*e*−08	+	MFMD	1.401*e*−10	+
		GIBBS			MEME		
	TBP	MFMD	2.2*e*−16	+	MFMD	2.200*e*−16	+
		GIBBS			MEME		

+ There is statistical difference (MFMD better); = There is no difference; - There is statistical difference (MFMD worse)

This is even more visible in smaller datasets, as shown in Table 4, where the MFMD performed considerably better than MEME and Gibbs Motif Sampler. The MCB, PDR3 and NF-Kb are the smallest real datasets, having 6, 7 and 6 sequences respectively. MFMD ties with Gibbs Motif Sampler in NF-Kb and wins both in the others. In this context, with less number of samples, the estimation of the probabilistic model loses precision, but MFMD was able to recognize a greater number of motifs. In general, the best performance achieved by MFMD can be attributed to its optimization architecture and the most effective way that its heuristics are applied, allowing to explore the search space more efficiently and thus achieving better results.

Table 7 shows the result of the statistical test performed with the f-scores obtained by each approach. The following experiments were conducted: MFMD vs Gibbs Motif Sampler and MFMD vs MEME and the results were presented as follows: (+) there is statistical difference favorable to MFMD; (=) there is no statistical difference; and (-) There is statistical difference unfavorable to MFMD. The statistical test corroborates the results presented in Table 6 (ranking) where MFMD obtained an advantage in relation to the other approaches.

MFMD uses in construction step *q*=0.9. Whether *q*=1 then the algorithm is greedy. On the other hand, whether *q*=0, then the algorithm is random. While low values of *q* promote randomness and consequently low-quality solutions, high values of *q* lead the algorithm to local optima.

The same is true of RCL. If *RCL*=1, then the algorithm becomes greedy, even though *q*=0. Conversely, if *RCL*=*L*−*w*+1, the algorithm becomes random. It is important to note that in both cases a compromise must be found between randomness and greediness.

The significance level used in the Pattern Matching step was 0.0001. The addition of this value would lead to greater permissibility to the method, increasing the number of predicted false positives. On the other hand, its decrease would leave the approach more “rigid” and consequently a smaller number of true positives would be observed. Therefore, the correct adjustment of this parameter directly implies the prediction quality of the algorithm.

Although all the programs compared in this work are based on probabilistic models, there are considerable differences in the results obtained due to the size of the search space and the existence of a large number of possible solutions. Optimization algorithms, such as MEME for example, can optimize the statistical models locally. However, the inherent multi-modality of the search space, in general, does not allow purely local driven optimization procedures to explore many different solutions. The MFMD architecture allows greater flexibility of search engine space because it applies an evolutionary process to a population of possible candidate solutions.

**Fig. 4:**
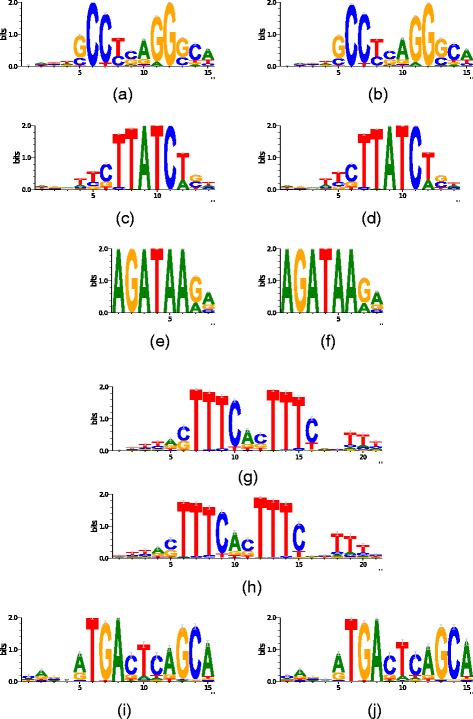
Comparison between real logos and logos found by MFMD in ChIP–seq datasets. **a** TFAP2A real Logo. **b** TFAP2A MFMD Logo. **c** GATA2 real Logo. **d** GATA2 MFMD Logo. **e** GATA3 real Logo. **f** GATA3 MFMD Logo. **g** IRF1 real Logo. **h** IRF1 MFMD Logo. **i** NFE2L2 real Logo. **j** NFE2L2 MFMD Logo

**Fig. 5:**
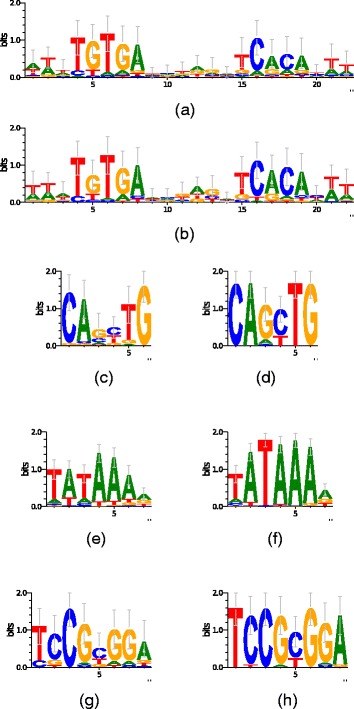
Comparison between real logos and logos found by MFMD in real datasets. **a** CRP real Logo. **b** CRP MFMD Logo. **c** MYOD real Logo. **d** MYOD MFMD Logo. **e** TBP real Logo. **f** TBP MFMD Logo. **g** PDR3 real Logo. **h** PDR3MFMD Logo

Finally, Figs. 4 and 5 compare the logos obtained by MFMD in the JASPAR and real datasets experiments with the logos generated from the real motifs. In them, we can see that the logos generated by the MFMD is very similar to the real logos.

## Conclusions

In this work we propose a new algorithm for the motif discovery in DNA sequences using local search and evolutionary algorithms as an optimization strategy.

The proposed approach, called MFMD, starts from a population of gradually generated motifs and performs an extensive search through operations such as recombination, mutation, and local search.

To demonstrate the efficiency of MFMD, several experiments were carried out in four groups of datasets: simulated datasets; JASPAR (datasets and motifs extracted from ChIP-seq experiments) and real datasets experiments. Through the comparisons made between the MFMD and other approaches found in the literature, it can be concluded that the MFMD was able to achieve better results in most of the experiments in all datasets.

Although there are several more robust probabilistic models than PSSM, such as Dinucleotide Weight Matrices (DWM) [37] and Transcription Factor Flexible Models (TFFM) [38], the objective of this work was to highlight the efficiency of the hybrid evolutionary approach in relation to approaches Literature.

In future works, we intend to investigate other forms of representation. While there is a considerable effort in the scientific community, it remains a complex challenge for computational biologists to predict convincing regulatory elements in DNA sequences.

Current paradigms of motif discovery can be seen as an approximation of biological reality, although recent efforts have sought to include correlation between motif positions [39], phylogenetic information [40], and synergistic relationships among transcription factors [41]. As the complexity of these models increases, the need arises to develop increasingly sophisticated algorithms that can find optimal solutions for these models and this will become increasingly important over time.

## Availability and requirements

**Project Name:** Sequence motif finder using memetic algorithm

**Project Home Page:** https://github.com/jadermcg/mfmd

**Operating System(s):** Linux Ubuntu 16.04 LTS

**Programming Language:** Java

**Other Requirements:** Java 8 (https://www.java.com/download/) or higher, Weblogo 3 (http://weblogo.threeplusone.com/), R 3.3.3 (https://cran.r-project.org/) or higher

**License:** GNU GPL

## Additional file

Additional file 1 Simulated datasets details. This file contains the description of the simulated datasets used in this paper. (PDF 114 kb)

## References

[1] D’haeseleer P (2006). What are DNA sequence motifs. Nat Biotechnol.

[2] Sandve GK, Drabløs F (2006). A survey of motif discovery methods in an integrated framework. Biology Direct.

[3] Wray GA, Hahn MW, Abouheif E, Balhoff JP, Pizer M, Rockman MV (2003). The evolution of transcriptional regulation in eukaryotes. Mol Biol Evol.

[4] Alberts B, Johnson A, J Lewis, Roberts K, Walter P (2007). Molecular biology of the cell.

[5] Das MK, Dai HK (2007). A survey of DNA motif finding algorithms. BMC Bioinformatics.

[6] D’haeseleer P (2006). How does DNA sequence motif discovery work?. Nat Biotechnol.

[7] Durbin R, Edy SR, Krogh A, Mitchison G (1998). Biological sequence analysis: Probabilistic models of proteins and nucleic acids.

[8] Bailey TL, Williams N, Misleh C, Li WW (2006). MEME: discovering and analyzing DNA and protein sequence motifs. Nucleic Acids Res.

[9] Hertz GZ, Stormo GD (1999). Identifying DNA and protein patterns with statistically significant alignments of multiple sequences. Bioinformatics.

[10] Neuwald AF, Liu JS, Lawrence CE (1995). Gibbs motif sampling: detection of bacterial outer membrane protein repeats. Protein Sci.

[11] Tompa M, Li N, Bailey TL, Church GM, De Moor B, Eskin E (2005). Assessing computational tools for the discovery of transcription factor binding sites. Nat Biotechnol.

[12] Sagot MF. Spelling approximate repeated or common motifs using a suffix tree. In: LATIN 98: Theoretical Informatics. Springer;1998. p. 374–390.

[13] Pavesi G, Mauri G, Pesole G (2001). An algorithm for finding signals of unknown length in DNA sequences. Bioinformatics.

[14] Moscato P, Norman MG (1992). A memetic approach for the traveling salesman problem implementation of a computational ecology for combinatorial optimization on message-passing systems. Parallel Comput Transputer Appl.

[15] Feo TA, Resende MG (1995). Greedy randomized adaptive search procedures. J Glob Optim.

[16] Hansen P, Mladenović N (2001). Variable neighborhood search: Principles and applications. Eur J Oper Res.

[17] Garbelini JC, Kashiwabara AY, Sanches DS. Discovery Motifs by Evolutionary Computation. In: Proceedings of the 2016 on Genetic and, Evolutionary Computation Conference Companion. Denver: ACM; 2016. p. 1463–1464.

[18] Garbelini JMC, Kashiwabara AY, Sanches DS. Discovery Biological Motifs Using Heuristics Approaches. In: Intelligent Systems (BRACIS), 2016 5th Brazilian Conference on. Recife: IEEE; 2016. p. 175–180.

[19] Li M, Ma B, Wang L. Finding similar regions in many strings. In: Proceedings of the thirty-first annual ACM symposium on Theory of computing. Atlanta: ACM; 1999. p. 473–482.

[20] Stormo GD, Hartzell GW (1989). Identifying protein-binding sites from unaligned DNA fragments. Proc Natl Acad Sci.

[21] Fogel GB, Weekes DG, Varga G, Dow ER, Harlow HB, Onyia JE (2004). Discovery of sequence motifs related to coexpression of genes using evolutionary computation. Nucleic Acids Res.

[22] Zia A, Moses AM (2012). Towards a theoretical understanding of false positives in DNA motif finding. BMC Bioinformatics.

[23] Wasserman WW, Sandelin A (2004). Applied bioinformatics for the identification of regulatory elements. Nat Rev Genet.

[24] Tatusov R, Lipman D. Dust, in the NCBI. Toolkit available at ftp://ftp.ncbi.nlm.nih.gov/pub/agarwala/dustmasker/.

[25] Benos PV, Bulyk ML, Stormo GD (2002). Additivity in protein–DNA interactions: how good an approximation is it?. Nucleic Acids Res.

[26] Stormo GD (2000). DNA binding sites: representation and discovery. Bioinformatics.

[27] British Society for the Philosophy of Science and British Society for the History of Science. The British journal for the philosophy of science. vol. 1.Oxford: Aberdeen University Press; 1950.

[28] Sandelin A, Alkema W, Engström P, Wasserman WW, Lenhard B (2004). JASPAR: an open-access database for eukaryotic transcription factor binding profiles. Nucleic Acids Res.

[29] Blanco E, Farre D, Alba MM, Messeguer X, Guigo R (2006). ABS: a database of Annotated regulatory Binding Sites from orthologous promoters. Nucleic Acids Res.

[30] Zhu J, Zhang MQ (1999). SCPD: a promoter database of the yeast Saccharomyces cerevisiae. Bioinformatics.

[31] Thompson W, Rouchka EC, Lawrence CE (2003). Gibbs Recursive Sampler: finding transcription factor binding sites. Nucleic Acids Res.

[32] Shaw WM, Burgin R, Howell P (1997). Performance standards and evaluations in IR test collections: Cluster-based retrieval models. Inf Process Manag.

[33] Kuncheva LI, Rodríguez JJ. An experimental study on rotation forest ensembles. In: Multiple Classifier Systems. Prague: Springer; 2007. p. 459–468.

[34] Shapiro SS, Wilk MB (1965). An analysis of variance test for normality (complete samples). Biometrika.

[35] Mankiewicz R. The story of mathematics. Cassell. 2000.

[36] Wilcoxon F (1945). Individual comparisons by ranking methods. Biom Bull.

[37] Siddharthan R (2010). Dinucleotide weight matrices for predicting transcription factor binding sites: generalizing the position weight matrix. PLoS ONE.

[38] Mathelier A, Wasserman WW (2013). The next generation of transcription factor binding site prediction. PLoS Comput Biol.

[39] Zhou Q, Liu JS (2004). Modeling within-motif dependence for transcription factor binding site predictions. Bioinformatics.

[40] Siddharthan R, Siggia ED, Van Nimwegen E (2005). PhyloGibbs: a Gibbs sampling motif finder that incorporates phylogeny. PLoS Comput Biol.

[41] Gupta M, Liu JS (2005). De novo cis-regulatory module elicitation for eukaryotic genomes. Proc Natl Acad Sci U S A.

