# Supplementary material for: Sequence motif finder using memetic algorithm
Source: BMC Bioinformatics. 2018 Jan 3;19:4. doi: 10.1186/s12859-017-2005-1 (PMC5751424; doi:10.1186/s12859-017-2005-1)
Supplement: Additional file 1 — Simulated datasets details. This file contains the description of the simulated datasets used in this paper. (PDF 114 kb) [file 12859_2017_2005_MOESM1_ESM.pdf]

Additional File 1
Datasets details

In simulated data we have generated 300 synthetic datasets divided into groups according to 3 factors: dataset size, motif length and motif conservation level. Six different groups were created that mix these 3 factors (illustrated on Table 1) with 50 datasets each. The background probability used to compile these datasets was as follows: $A\left(\frac{1}{10}\right)$, $C\left(\frac{2}{10}\right)$, $G\left(\frac{3}{10}\right)$ and $T\left(\frac{4}{10}\right)$. The algorithm used to build simulated datasets can be downloaded at `https://github.com/jadermcg/buildingDatasets`.

Table 2 shows the results obtained by the predictors in each group of the simulated datasets. Therefore, for MFMD approach, the value presented refers to the mean of the averages (30 runs $\times$ 50 datasets), while for MEME and Gibbs Motif Sampler the value is the average of the best executions of each approach.

Table 3 shows the results obtained by the approaches in the ranking analysis in simulated datasets and in Table 4 all approaches are ordered according to the performance obtained in Table 3. In all groups MFMD was able to perform better than MEME and Gibbs Motif Sampler. Also, it is interesting to note that MEME has found a good performance only in group 4, reaching 17 wins while MFMD had 18 wins. Gibbs Motif Sampler was not able to perform well getting the worst results in all simulated datasets.

Finally, Table 5 shows the results of the statistical test performed with the f-scores obtained by each approach in simulated datasets.

Table 1. Summary of synthetic datasets.

Group	Number of sequences	Motif size	Conservation level
1	100	20	90%
2	100	20	65%
3	100	10	80%
4	30	20	90%
5	30	20	65%
6	30	10	80%

Table 2. Results achieved by predictors in simulated datasets.

Group	Predictor	Precision	Recall	F-Score
1	MFMD	0.995 ± 0.007	0.998 ± 0.005	0.996 ± 0.009
	MEME	0.993 ± 0.007	0.990 ± 0.009	0.991 ± 0.013
	GIBBS	0.978 ± 0.007	0.958 ± 0.009	0.967 ± 0.011
2	MFMD	0.856 ± 0.041	0.866 ± 0.043	0.860 ± 0.039
	MEME	0.839 ± 0.033	0.833 ± 0.039	0.835 ± 0.035
	GIBBS	0.795 ± 0.047	0.796 ± 0.041	0.795 ± 0.037
3	MFMD	0.961 ± 0.019	0.968 ± 0.029	0.964 ± 0.011
	MEME	0.931 ± 0.023	0.937 ± 0.017	0.933 ± 0.029
	GIBBS	0.769 ± 0.039	0.761 ± 0.053	0.764 ± 0.041
4	MFMD	0.995 ± 0.011	0.999 ± 0.015	0.996 ± 0.017
	MEME	0.991 ± 0.011	0.996 ± 0.015	0.993 ± 0.021
	GIBBS	0.975 ± 0.033	0.980 ± 0.017	0.977 ± 0.025
5	MFMD	0.809 ± 0.077	0.815 ± 0.081	0.811 ± 0.099
	MEME	0.787 ± 0.065	0.781 ± 0.081	0.783 ± 0.073
	GIBBS	0.751 ± 0.079	0.757 ± 0.067	0.753 ± 0.089
6	MFMD	0.969 ± 0.033	0.964 ± 0.051	0.966 ± 0.027
	MEME	0.918 ± 0.047	0.921 ± 0.051	0.919 ± 0.033
	GIBBS	0.722 ± 0.105	0.728 ± 0.107	0.724 ± 0.115

Table 3. Wins and losses in the simulated datasets organized by group.

Predictor	Group	Wins	Losses	Total
MFMD	1	56	14	42
	2	76	16	60
	3	91	7	84
	4	25	7	18
	5	57	30	27
	6	85	3	82
MEME	1	46	18	28
	2	59	39	11
	3	57	41	16
	4	22	5	17
	5	47	38	9
	6	51	36	15
GIBBS	1	12	83	–71
	2	18	126	–108
	3	0	100	–100
	4	4	39	–35
	5	26	62	–36
	6	1	98	–97

Table 4. Ranking of predictors in simulated datasets according to Table 3 (from best to worst).

Group	Predictors		
1	MFMD	MEME	GIBBS
2	MFMD	MEME	GIBBS
3	MFMD	MEME	GIBBS
4	MFMD	MEME	GIBBS
5	MFMD	MEME	GIBBS
6	MFMD	MEME	GIBBS

Table 5. Statistical test between MFMD vs GIBBS and MFMD vs MEME approaches. + There is statistical difference (MFMD better); = There is no difference; - There is statistical difference (MFMD worse).

Type	Group / Dataset	Approach	P-value	Result	Approach	P-value	Result
Synthetic	Group 1/24	MFMD GIBBS	$4.661e-09$	+	MFMD MEME	$3.577e-4$	+
	Group 1/40	MFMD GIBBS	$1.585e-04$	+	MFMD MEME	$1.350e-11$	-
	Group 2/23	MFMD GIBBS	$1.475e-12$	+	MFMD MEME	$2.200e-16$	+
	Group 2/38	MFMD GIBBS	0.65	=	MFMD MEME	$4.304e-12$	+
	Group 3/21	MDMF GIBBS	$2.2e-16$	+	MFMD MEME	$2.670e-05$	+
	Group 3/46	MFMD GIBBS	$2.2e-16$	+	MFMD MEME	$2.200e-16$	+
	Group 4/18	MFMD GIBBS	$1.792e-08$	+	MFMD MEME	$2.200e-16$	+
	Group 4/43	MFMD GIBBS	$2.127e-04$	-	MFMD MEME	$2.004e-12$	-
	Group 5/12	MFMD GIBBS	$2.2e-16$	+	MFMD MEME	$2.200e-16$	+
	Group 5/38	MFMD GIBBS	$2.2e-16$	+	MFMD MEME	$2.200e-16$	+
	Group 6/33	MFMD GIBBS	$2.2e-16$	+	MFMD MEME	$2.200e-16$	+
	Group 6/25	MFMD GIBBS	$2.2e-16$	+	MFMD MEME	$2.200e-16$	+
